# Case Report: A Novel Missense Variant in the *SIPA1L3* Gene Associated With Cataracts in a Chinese Family

**DOI:** 10.3389/fgene.2021.715599

**Published:** 2021-09-16

**Authors:** Duo Yang, Haiyan Zhou, Jiwu Lin, Shuangxi Zhao, Hao Zhou, Zhaochu Yin, Bin Ni, Yong Chen, Wanqin Xie

**Affiliations:** ^1^Department of Ophthalmology, The Jili Hospital of Liuyang and the Eye Hospital of Liuyang, Changsha, China; ^2^National Health Committee Key Laboratory of Birth Defects for Research and Prevention, Hunan Provincial Maternal and Child Health Care Hospital, Changsha, China; ^3^State Key Laboratory of Medicinal Chemical Biology, Tianjin Key Laboratory of Protein Science, College of Life Sciences, Nankai University, Tianjin, China

**Keywords:** Rap GTPase, GTPase-activating protein, *SIPA1L3*, RapGAP, cataract

## Abstract

The signal-induced proliferation-associated 1-like 3 (*SIPA1L3*) gene that encodes a putative Rap GTPase-activating protein (RapGAP) has been associated with congenital cataract and eye development abnormalities. However, our current understanding of the mutation spectrum of *SIPA1L3* associated with eye defects is limited. By using whole-exome sequencing plus Sanger sequencing validation, we identified a novel heterozygous c.1871A > G (p.Lys624Arg) variation within the predicted RapGAP domain of SIPA1L3 in the proband with isolated juvenile-onset cataracts from a three-generation Chinese family. In this family, the proband's father and grandmother were also heterozygous for the c.1871A > G variation and affected by cataracts varying in morphology, severity, and age of onset. Sequence alignment shows that the Lys 624 residue of SIPA1L3 is conserved across the species. Based on the resolved structure of Rap1–Rap1GAP complex, homology modeling implies that the Lys 624 residue is structurally homologous to the Lys 194 of Rap1GAP, a highly conserved lysine residue that is involved in the interface between Rap1 and Rap1GAP and critical for the affinity to Rap·GTP. We reasoned that arginine substitution of lysine 624 might have an impact on the SIPA1L3-Rap·GTP interaction, thereby affecting the regulatory function of SIPA1L3 on Rap signaling. Collectively, our finding expands the mutation spectrum of *SIPA1L3* and provides new clues to the molecular mechanisms of *SIPA1L3*-related cataracts. Further investigations are warranted to validate the functional alteration of the p.Lys624Arg variant of SIPA1L3.

## Introduction

Cataract, clinically characterized by opacity in the ocular lens, is a common eye disease that may cause vision impairment or blindness. Congenital cataract is usually diagnosed in the first year of life, with an average prevalence of 72 per 100,000 births worldwide (Shiels and Hejtmancik, 2019). About 30% of congenital cataract cases have a genetic etiology (Li et al., 2020). The most common inheritance pattern of congenital cataract is autosomal dominant, but it can be autosomal recessive or X-linked inheritance in some cases (Berry et al., 2020). Notably, while most inherited cataracts present at birth or early childhood, some can have an onset as late as adulthood (Shiels and Hejtmancik, 2019). More than 40 genes have been associated with congenital/inherited cataract. These genes mainly encode crystallins, membrane proteins, transcription or developmental factors, cytoskeletal proteins, and other proteins in the lens (Shiels and Hejtmancik, 2019; Berry et al., 2020).

The signal-induced proliferation-associated 1-like 3 (*SIPA1L3*) gene [Online Mendelian Inheritance in Man (OMIM) ^*^616655 and #616851], located on chromosome 19, encodes a putative GTPase-activating protein (GAP) specific for the small GTPases of Rap family. The full-length human SIPA1L3 protein consists of 1,781 amino acid residues, with the predicted Rap GTPase-activating protein (RapGAP) domain (residues 611–828), PDZ domain (residues 966–1,042), and C-terminal coiled–coiled domain (residues 1,720–1,774). The RapGAP domain of the SIPA1L3 homolog SIPA1L1 has been shown to interact with Rap1, facilitating the switch of Rap1 from an active GTP-bound state to an inactive GDP-bound state (Evers et al., 2015).

Rap signaling plays crucial roles in cell growth, differentiation, and organization of the cytoskeleton in the human lens (Spilker and Kreutz, 2010). Hence, mutations in *SIPA1L3* may disturb the regulation of Rap signaling during embryonic eye development and cause eye defects (Greenlees et al., 2015). *SIPA1L3* was first identified as a causative gene for congenital cataract in a distantly consanguineous German family with two sisters homozygous for the c.4489C > T (p.R1497X) non-sense mutation (Evers et al., 2015). This mutation results in a truncated protein lacking the last 284 amino acids and is thought to be loss of function. In another independent study, two unrelated patients who carried distinct mutations in *SIPA1L3* were documented. Patient 1 who manifested severe lens and ocular anterior segment abnormalities with cataracts carried a *de novo* balanced translocation, 46,XY,t(2;19)(q37.3;q13.1), which generated a variant of *SIPA1L3* with a transection of 5′ un-translated region. Reduced *SIPA1L3* expression was detected in the patient's lymphoblast. Patient 2 from the same study was found with bilateral congenital cataracts and heterozygous for a missense variation c.442G > T (p.Asp148Tyr). The p.Asp148Tyr mutation resides in the putative actin-binding domain of SIPA1L3, and the mutant has been shown to cause abnormal cytoskeleton organization and cell adhesion in epithelial cells (Greenlees et al., 2015).

Though *SIPA1L3* has been known as a causative gene for cataracts and eye anterior dysgenesis, overall, our current understanding of the mutation spectrum of *SIPA1L3* related to eye defects is limited. In this study, we report a novel missense variant, namely, the *SIPA1L3* c.1871A > G (p.Lys624Arg) variant that co-segregated with cataracts in a three-generation Chinese family.

## Materials and Methods

### Ethical Compliance

This study was approved by the Ethics Committee of Hunan Provincial Maternal and Child Health Care Hospital. Written informed consent was obtained from the patient or the patient's legal representative for genetic tests.

### Patients

A 17-year-old male presented complaining of loss of vision of the left eye. At the age of 14, he sensed a gradual blurring of vision of the left eye with fixed central visual occlusion, and he left it untreated at that time. The symptoms of the left eye became aggravated when he aged 15. At the age of 17, he incidentally found that the left eye had lost vision. He visited the ophthalmic outpatient at the Eye Hospital of Liuyang with his grandmother. Physical examination showed that the patient had normal cardiopulmonary function with no remarkable congenital anomalies or developmental disorders. Opacity of the lens in the left eye was readily visible. Hand movement test, count finger test, and light perception of the left eye were passed by the patient, and color vision was normal. Distant vision of the right eye was 20/80, and near vision of the right eye was 0.5/33 cm. The corneas of both eyes were transparent and iris texture was clear. No abnormality was seen in routine blood tests. During the outpatient visit, the patient's grandmother (aged 71) reported that she used to suffer from severe cataracts and had received surgery in the hospital and that the patient's father (aged 40) also had cataracts, but his vision in both eyes was still acceptable and had not received treatment yet. The index patient was treated by cataract surgery. Vision acuity examination after surgery showed distant vision 20/100 and near vision 0.7/33 cm for the left eye and no vision change for the right eye. To find out the potential causative mutation for cataract in the family, the index patient and his parents and grandmother volunteered for genetic tests.

### Genetic Analysis

Genomic DNA extraction from blood was performed using QIAamp DNA Blood Mini Kit (QIAGEN, Hilden, Germany). Chromosomal microarray analysis (CMA) was performed using Affymetrix CytoScan®750 K Array (Affymetrix Inc., CA, USA) according to the manufacturer's instructions.

Whole-exome sequencing (WES) was performed following the pipeline protocols at BGI Clinical Testing Center (Shenzhen, Guangdong, China). In this clinic-oriented service, the annotation of sequence variants including single-nucleotide variation (SNV) and insertion–deletion (InDel) was limited to those derived from the definitive disease-associated genes in the OMIM database. Among the annotated variants, further filtering for identification of candidate variants was based on phenotypic overlap with genes in OMIM; minor allele frequency [<1% in public databases including NHLBI GO Exome Sequencing Project (ESP6500), 1000 Genomes, Exome Aggregation Consortium (ExAC), Genome Aggregation Database (GnomAD), and/or GnomAD-EAS]; and predictive results from bioinformatics tools including SIFT, Mutation Taster, and PolyPhen-2. Sanger sequencing was conducted at RuiBiotech (Beijing, China), and the PCR primers were designed according to the reference genomic sequence of *SIPA1L3* (GenBank accession number: NG_046730.1).

### Molecular Modeling

In terms of the structural data of Rap1–Rap1GAP complex deposited in the protein data bank (PDB entry: 1SRQ), homology modeling of the RapGAP domain (residues 611 to 828) of SIPA1L3 (UniProKB: O60292) was performed using the SWISS-MODEL (https://swissmodel.expasy.org/) (Waterhouse et al., 2018). All structure figures were prepared using PyMOL (http://pymol.sourceforge.net/). The schematic diagram of Rap1–Rap1GAP interactions was generated by using LigPlus (http://www.ebi.ac.uk/thornton-srv/software/LigPlus/) (Laskowski and Swindells, 2011).

## Results

### Characteristics of the Cataracts in the Index Patient

**Figure 1:**
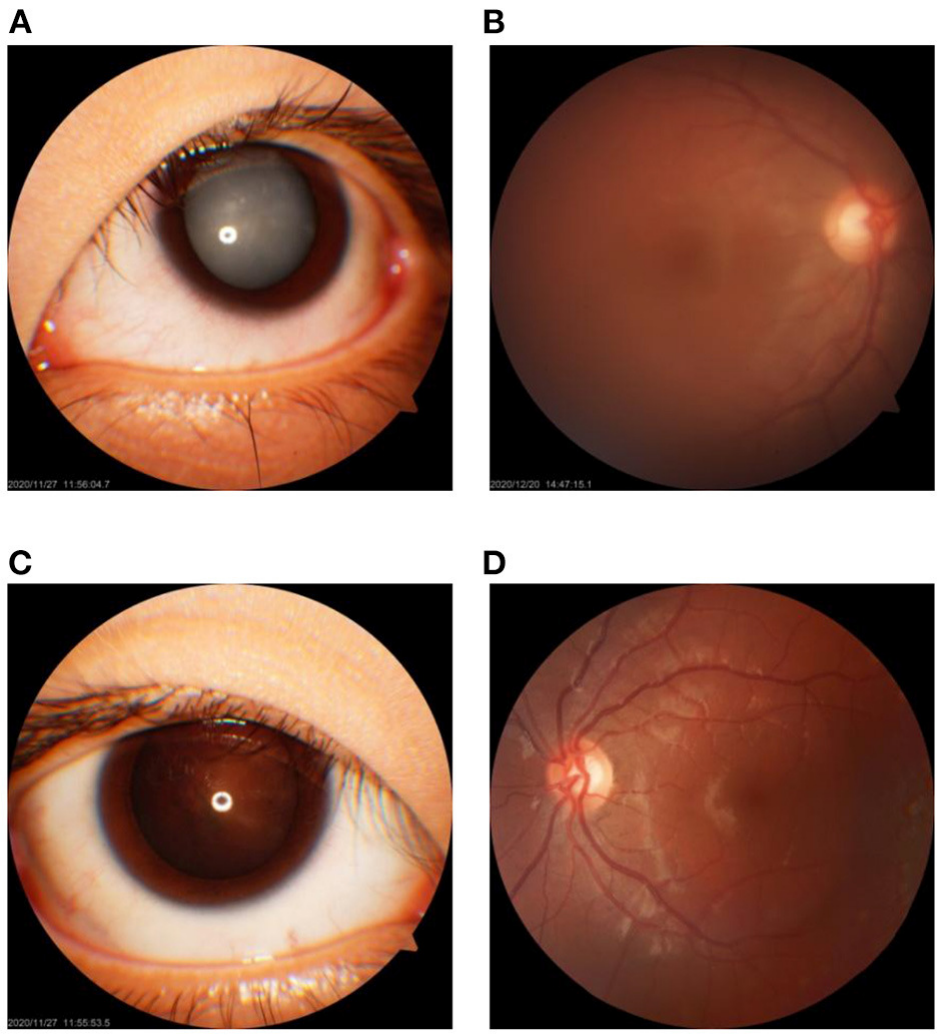
Clinical feature of the cataracts in the 17-year-old male proband. **(A,B)** The cortical cataract of the lens and the fundus of the left eye, respectively. **(C,D)** The nuclear cataract of the lens of the right eye with cloudiness at the posterior lens capsule and the fundus of the right eye, respectively.

The left eye of the index patient presented with cortical and nuclear white opacities of the lens (Figure 1A). The lens posterior capsule and the fundus of the left eye could not be seen clearly (Figure 1B). His right eye showed gray–white opacities at the Y suture region of the lens nucleus, slight deposition of granular cloudy substances at the lens posterior capsule, and clear optic disc in fundus (Figures 1C,D). During the cataract surgery, slight liquefaction of the lens cortex and softness in the lens nucleus of the right eye were noticed.

### Genetic Variation

**Figure 2:**
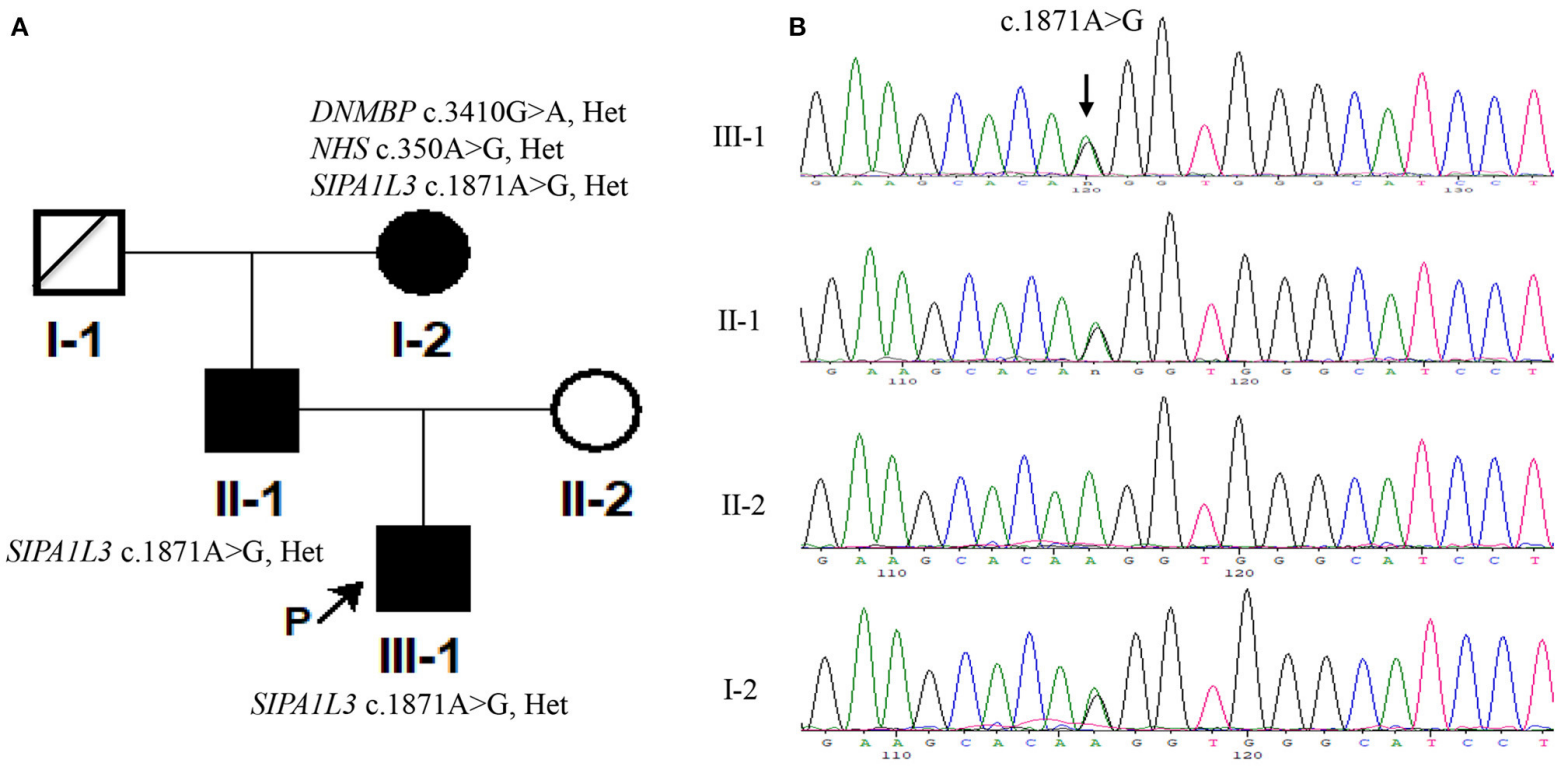
The signal-induced proliferation-associated 1-like 3 (*SIPA1L3*) c.1871A > G variation in the patients was detected by using Sanger sequencing. **(A)** The pedigree of the Chinese family in the current study. **(B)** Sanger sequencing results of c.1871A > G variation in the Chinese family. The genomic DNA used for PCR amplification was extracted using peripheral blood (III-1 and I-2) or oral swabs (II-1 and II-2). The sequencing was performed in both forward and reverse direction on a genetic analyzer 3730XL (Applied Biosystems). The results of forward sequencing are representatively shown.

CMA detected no common pathogenic copy number variations in the index patient and his grandmother (III-1 and II-2, Figure 2A) (data not shown). However, the index patient carried a heterozygous c.1871A > G/p.Lys624Arg variation in *SIPA1L3* that is thought to be associated with cataract as revealed by WES (Supplementary Data Sheet 1). This variation was also detected in his grandmother (II-2) with two additional heterozygous variations related to cataracts, namely, the *DNMBP* c.3410G > A/p.Arg1137Gln on chromosome 10 and the *NHS* c.350C>G/p.Ala117Gly on chromosome X (Supplementary Data Sheet 2). Considering the previous findings that the *DNMBP* and *NHS* are associated with autosomal recessive infantile cataract (Ansar et al., 2018) and X-linked congenital cataract (Burdon et al., 2003; Ling et al., 2019), respectively, and the fact that the *DNMBP* c.3420G > A and *NHS* c.350C > G variations were absent from the index patient, we thus chose to focus on the *SIPA1L3* c.1871A > G variation for further analysis thereafter.

The *SIPA1L3* c.1871A > G missense variation possessed an allele frequency of 0.000009 in GnomAD (http://gnomad.broadinstitute.org) and was predicted to be deleterious by using SIFT (http://sift.jcvi.org) and Mutation Taster (http://www.mutationtaster.org). Sanger sequencing confirmed that the index patient and his father and grandmother (III-1, II-1, and I-2; Figure 2B) were heterozygous for the c.1871A > G variation and that this variation was absent from his mother (II-2, Figure 2B) and 200 unrelated controls comprising of 100 couples for assisted reproduction technology in Hunan Provincial Maternal and Child Health Care Hospital (data not shown). Moreover, the *DNMBP* c.3420G > A and *NHS* c.350C > G variations identified in the grandmother (I-2) were not detected in the index patient (III-1) and his father (II-1) (Figure 2A). According to ACMG guidelines (Richards et al., 2015), rules PM2, PP1, and PP3 were applied, and the c.1871A > G variant was considered to be of uncertain significance.

### Molecular Modeling

**Figure 3:**
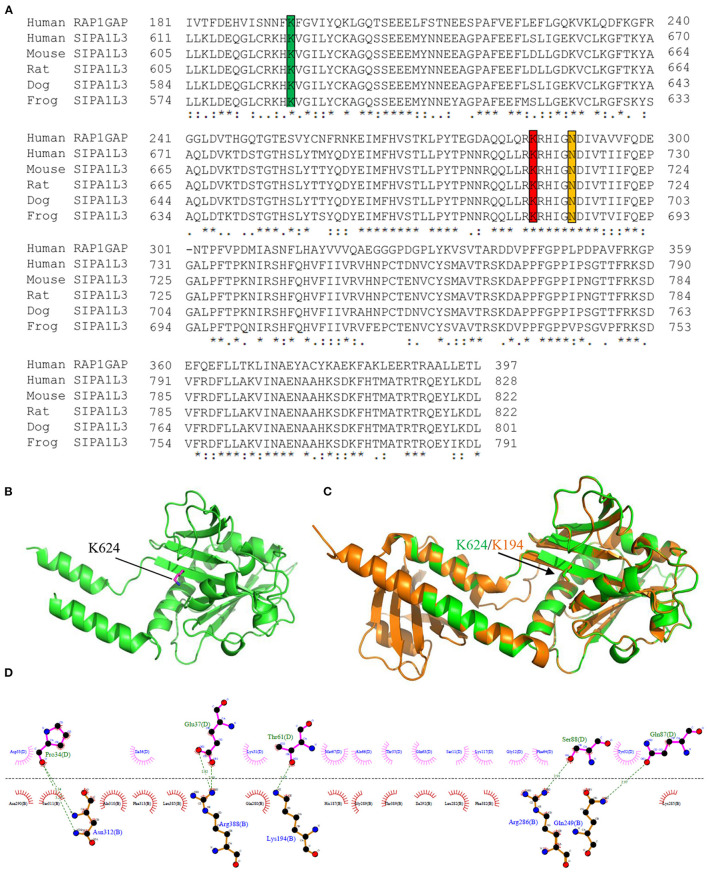
The lysine residue 624 within the Rap GTPase-activating protein (RapGAP) domain of SIPA1L3 is highly conserved and shares homology with the lysine residue 194 of human Rap1GAP. **(A)** Alignment of RapGAP domain of SIPA1L3 of different species with the human Rap1GAP. The highly conserved lysine residues 194 and 285 and the key catalytic asparagine residue (Asn) 290 of Rap1GAP are marked by green, red, and yellow, respectively. **(B)** Homology modeling shows the conformation of Lys 624 (purple) within the RapGAP domain of SIPA1L3. **(C)** The Lys 624 (green) is superimposed over the Lys 194 of Rap1GAP (brown). The crystal structure data of Rap1GAP was retrieved from Protein Data Bank (PDB Entry: 1SRQ). **(D)** In terms of the structural data of Rap1–Rap1GAP complex (1SRQ), the interface between Rap1 (molecule B) and Rap1GAP (molecule D) that involves the Lys 194 was generated using LigPlus software.

Sequence alignment reveals that the RapGAP domain (residues 611–828) of SIPA1L3 is highly conserved across the species and highly homologous to the RapGAP domain (residues 181–397) of human Rap1GAP (UniProKB: P47736) (Figure 3A). Molecular modeling indicates that the Lys 624 of SIPA1L3 is structurally homologous to the Lys 194 of Rap1GAP (Figures 3B,C). In terms of the resolved structure, the Lys 194 of Rap1GAP is involved in the interface between Rap1 and Rap1GAP, where the Lys 194 of Rap1GAP forms a non-covalent bond with the threonine residue 61 (Thr61) in the switch II region of Rap1 (Figure 3D). Based on homology, it is speculated that arginine substitution of Lys 624 of SIPA1L3 may have a potential impact on the SIPA1L3–Rap interaction.

## Discussion

SIPA1L3 is regarded as a negative regulator of Rap signaling that plays important roles in neuronal function and lens development (Spilker and Kreutz, 2010; Dolnik et al., 2016; Rothe et al., 2017). Previous studies have demonstrated that homozygous or heterozygous mutations in the *SIPA1L3* gene may cause isolated cataracts or eye development abnormalities including microphthalmia and anterior segment dysgenesis with cataracts (Evers et al., 2015; Greenlees et al., 2015). Of note, the reported *SIPA1L3*-related cataracts were characterized by an infantile or early onset with high severity (Table 1). The present study reports the co-segregation of SIPA1L3 Lys624Arg mutation with isolated cataracts in a three-generation Chinese family. The 17-year-old male proband and his father and grandmother were affected by cataracts varying in morphology, severity, and age of onset. Even within the proband, the development of cataracts in his two eyes was asymmetric (Table 1). Though the detailed ages of onset could not be identified for the father and grandmother in this Chinese family (II-1 and I-2 in the pedigree, Figure 2A), it is unlikely that their cataracts had a very early onset in terms of their description. Though the mutation status in the grandmother (I-2, Figure 2A) was complicated by the concomitant presence of missense variants in *DNMBP* and *NHS*, Sanger sequencing confirmed that the proband and his father (III-1 and II-1, Figure 2A) only carried the candidate variation *SIPA1L3* c.1871A > G (p.Lys624Arg). Based on the clinical findings in our study, the cataracts associated with SIPA1L3 p.Lys624Arg mutation can be heterogeneous within and between patients. As this kind of cataracts was found to affect the Y suture and the posterior lens capsule, central vision and development of visual system might be severely affected, leading to amblyopia. Early diagnosis and treatment may offer better vision and visual function to the affected individuals.

**Table 1:** Comparison of genotypes and phenotypes of patients with SIPA1L3-associated cataracts.

**Patients**	**Source**	**Mutation**	**Allele origin**	**Functional alteration**	**Age of onset**	**Symptoms**
The proband	This study	c.1871A>G/p.Lys624Arg, heterozygous	Inherited	Potential impact on interaction between SIPA1L3 and Rap1 (*in silico* analysis)	Initial onset at 14 years old and diagnosed at 17 years old	Left eye blindness with cortical and nuclear white cataract, and right eye with nuclear cataract
The sisters from a German consanguineous family	Evers et al., 2015	c.4489C>T/p.R1497X, homozygous	Inherited	No mRNA degradation, truncation of the last 284 amino acids	Diagnosed at 2 weeks old or soon after birth	Bilateral dense white cataracts
Patient 1	Greenlees et al., 2015	Balanced translocation 46,XY, t(2:19)(q37.3;q13.1)	*De novo*	Transection of the 5′ untranslated region, reduced protein expression in lymphoblast	Congenital	Bilateral microphthalmia and severe bilateral anterior segment dysgenesis characterized by sclerocornea, corneal-lens adhesion, cataracts and glaucoma, reduced anterior chamber depth bilaterally.
Patient 2	Greenlees et al., 2015	c.442G>T/p.Asp148Tyr heterozygous	Unknown	Abnormal cytoskeleton organization and cell adhesion	Congenital	Bilateral congenital cataracts
VCV001032651.1	ClinVar	c.947G>A/p.Arg316His	Unknown			Congenital cataract 45
VCV000931642.2	ClinVar	c.3535T>G/p.Tyr1179Asp	Unknown			Congenital cataract 45
VCV000931643.2	ClinVar	c.5248C>T/p.Arg1750Trp	Unknown			Congenital cataract 45

As implied by previous findings, the molecular mechanisms by which SIPA1L3 mutations cause cataracts could be diverse. The loss-of-function mechanism is implicated by the homozygous p.R1497X mutant identified in the two sisters with infantile bilateral dense white cataracts from a distantly consanguineous German family (Evers et al., 2015). The dominant-negative effect of the p.Asp148Tyr mutation in the putative actin-binding domain of SIPA1L3 is suggested by its heterozygosity in a patient with congenital bilateral cataracts, as well as the impact on cytoskeleton organization and cell adhesion upon transient expression of the mutant in epithelial cells (Greenlees et al., 2015). In addition, the possibility that haploinsufficiency may be causing cataracts could not be excluded. Reduced SIPA1L3 expression was detected in lymphoblast from a patient carrying a transaction of the 5′ untranslated region of *SIPA1L3* resulting from a balanced translocation. The patient manifested lens and ocular anterior segment abnormalities including cataracts (Greenlees et al., 2015). Regarding the p.Lys624Arg missense mutation in the present study, the co-segregation of this mutation with cataracts in the three-generation Chinese family implies an autosomal dominant inheritance. Interestingly, this mutation is localized in the predicted RapGAP domain of SIPA1L3, a region that is thought to confer the GAP activity of the protein. However, as the lysine residue at codon 624 is replaced by arginine (an amino acid with similar properties), prediction of functional consequence of this mutation is more challenging.

The RapGAP domain of SIPA1L3 shares homology with Rap1GAP, a GTPase-activating protein specific for the well-studied Rap1 (Spilker and Kreutz, 2010). The crystal structure of Rap1–Rap1GAP complex has been resolved (Scrima et al., 2008). We have found that the Lys 624 within the RapGAP domain of SIPA1L3 shares high similarity with Lys 194 of Rap1GAP, a residue that is positioned at the interface between Rap1 and Rap1GAP and interacts with threonine residue 61 (Thr 61) of Rap1 to stabilize the complex. Based on homology, we infer that Lys 624 is likely involved in the interaction between SIPA1L3 and Rap. Although both lysine and arginine are positively charged amino acids, arginine substitution of Lys 624 may have an impact on the SIPA1L3–Rap interaction in view of the steric structure. However, to test this hypothesis, whether there is a functional conservation between SIPA1L3 and Rap1GAP should be addressed prior to determining how the Lys624Arg mutation would affect the affinity between SIPA1L3 and Rap·GTP. Alternatively, arginine substitution may block the post-translational modification (e.g., acetylation or ubiquitination) that is supposed to add to the Lys 624. Again, this hypothesis remains to be experimentally validated.

In summary, we report a novel *SIPA1L3* missense variant, namely, the p.Lys624Arg mutant that co-segregated with isolated cataracts in a familial case. To the best of our knowledge, this is the first report regarding mutation in the putative RapGAP domain of SIPA1L3 that may be contributing to cataracts. Our finding expands the mutation spectrum of *SIPA1L3* and provides new insights into the pathogenesis of *SIPA1L3*-related cataracts.

## Data Availability Statement

The datasets for this article are not publicly available due to concerns regarding participant/patient anonymity. Requests to access the datasets should be directed to the corresponding author.

## Ethics Statement

The studies involving human participants were reviewed and approved by Ethics Committee of Hunan Provincial Maternal and Child Health Care Hospital. Written informed consent to participate in this study was provided by the participants' legal guardian/next of kin.

## Author Contributions

DY and SZ recruited the patients and noted the clinical phenotypes of the patients. HaiZ and JL performed the experiments. HaoZ performed molecular modeling. ZY and BN interpreted the data of genetic tests. WX and YC designed the study and reviewed all the data. WX wrote the manuscript daft. All authors contributed to the article and approved the submitted version.

## Funding

This work was supported by grants from the Health Committee of Hunan Province (C20180704 and 225 Talents Project), the Natural Science Foundation of Hunan Province (2019JJ80078), and the Major Scientific and Technological Projects for Collaborative Prevention and Control of Birth Defects in Hunan Province (2019SK1010).

## Conflict of Interest

The authors declare that the research was conducted in the absence of any commercial or financial relationships that could be construed as a potential conflict of interest.

## Publisher's Note

All claims expressed in this article are solely those of the authors and do not necessarily represent those of their affiliated organizations, or those of the publisher, the editors and the reviewers. Any product that may be evaluated in this article, or claim that may be made by its manufacturer, is not guaranteed or endorsed by the publisher.
